# Early Type 2 diabetes risk prediction using explainable machine learning in a two-stage approach

**DOI:** 10.3389/fdgth.2026.1743619

**Published:** 2026-03-27

**Authors:** Silas Majyambere, Tony Lindgren, Celestin Twizere, Isaac Ntakirutimana

**Affiliations:** 1 Department of Computer and Systems Sciences, Stockholm University, Stockholm, Sweden; 2 Centre of Excellence in Biomedical Engineering and E-Health (CEBE), University of Rwanda, Kigali, Rwanda

**Keywords:** diabetes management, diabetes prediction, explainable machine learning, interpretability, Multi-Layer Perceptron, shap

## Abstract

**Background:**

Diabetes is a chronic disease characterized by elevated blood glucose levels. Without early detection and proper management, it can lead to serious complications and increase healthcare costs. Its global prevalence is rising, with many cases remaining undiagnosed. In this study, we developed an explainable machine learning model using a two-stage approach for predicting diabetes.

**Methods:**

Five machine learning (ML) models, including Multi-Layer Perceptron, Support Vector Machine, K-Nearest Neighbor, Extreme Gradient Boosting (XGBoost), and Naïve Bayes, were trained and evaluated using a two-stage approach. In Stage one, a public dataset containing 520 samples was used, and Shapley Additive exPlanations (SHAP) and MLP weights were applied for feature selection. In Stage two, the same models were trained and evaluated using a dataset of 270,943 samples collected from Rwanda. SHAP was further employed to explain the model output.

**Results:**

In Stage one, the Multi-Layer Perceptron model achieved the best performance on a public dataset, with an accuracy of 95.19%. Feature selection techniques identified the top 10 influential predictors associated with diabetes risk, including those recommended by diabetes care providers in Rwanda. In Stage two, the XGB model outperformed other models, achieving an accuracy of 97.14%.

**Conclusion:**

This study presents a two-stage, explainable machine learning framework for systematic screening for type 2 diabetes. The first stage evaluates risk based on reported symptoms, while the second stage incorporates demographic, anthropometric, and vital sign data for refined risk assessment. Integration of these models into the mUzima mobile application can enhance community health workers' capacity to identify and refer high-risk individuals. By enabling early and accurate detection, the proposed approach has the potential to reduce undiagnosed diabetes and support improved disease management.

## 1 Introduction

Diabetes is a chronic, non-communicable disease characterized by elevated blood glucose levels. Its prevalence continues to increase globally. According to the International Diabetes Federation (IDF) report (1), there were 589 million people living with diabetes worldwide in 2024, with projections estimating this number to rise to 853 million by 2050. Early detection and effective blood glucose management remain the most crucial strategies for controlling diabetes. Undiagnosed cases (1) pose a serious risk to both patients and healthcare providers, as individuals often seek the screening of Type 2 Diabetes Mellitus (T2DM) only after developing symptoms related to complications. These complications could be prevented through timely diagnosis and treatment.

In Rwanda, initiatives to increase awareness of non-communicable diseases (NCDs), including diabetes, have been strengthened through nationwide campaigns led by Community Health Workers (CHWs), also referred to as home-based care practitioners (2). These CHWs are trained volunteers without formal medical education who receive targeted instruction and ongoing support from the Ministry of Health (MoH) to identify and manage conditions such as malaria, diabetes, and hypertension. Using smartphones, CHWs collect patient data through a mobile Health (mHealth) application called mUzima (3), which is integrated with OpenMRS, an electronic health records system used by most public health centers, enabling efficient data capture and real-time patient monitoring. To increase awareness in urban communities, Rwanda has introduced a community sports initiative known as “Car Free Day” (4), during which city residents participate in a collective marathon. After the event, participants are offered free screenings for non-communicable diseases, including diabetes. These initiatives are crucial in addressing the issue of undiagnosed diabetes; however, participation remains low, underscoring the need for technology-driven approaches to diabetes screening**.**

Machine learning has demonstrated significant potential in accurately identifying individuals at risk of developing Type 2 Diabetes Mellitus (T2DM). In (5), a LightGBM model achieved an accuracy of 91.47% when applied to the Pima Indian Diabetes dataset. Similarly, the study in (6) proposed a hybrid machine learning model for T2DM prediction, with experimental results indicating that Logistic Regression outperformed other algorithms, achieving an accuracy of 99.34%. High predictive performance was also observed in (7), where a Random Forest model was developed using an imbalanced dataset of 4,000 samples. After applying the Synthetic Minority Oversampling Technique (SMOTE) to address class imbalance, the model attained an accuracy of 95.12%.

Despite the high accuracy achieved by many machine learning models in diabetes prediction, their adoption in clinical settings remains limited due to the “black-box” nature of most algorithms, which lack interpretability. To overcome this limitation, the study in (8) employed both Shapley Additive Explanations (SHAP) and Local Interpretable Model-Agnostic Explanations (LIME) to interpret the predictions of a Logistic Regression model that achieved 86% accuracy in predicting T2DM using a dataset of 253,680 samples. This approach provided both global model explanations and individualized prediction insights. In (9), LIME was used to explain the predictions of an XGBoost model trained on the Pima Indian Dataset and a private dataset from Bangladesh, achieving an accuracy of 81% in identifying diabetes risk. The model was subsequently integrated into a mobile application to support clinical workflows. Similarly, in (10), SHAP was applied to interpret an ensemble model combining Logistic Regression and Adaptive Boosting, which was trained and validated on large-scale datasets and achieved an accuracy of 72.6% in predicting diabetes risk.

Despite a strong political commitment to addressing non-communicable diseases, including diabetes, Rwanda continues to report a high number of undiagnosed diabetes cases (11). Building upon existing national initiatives, this study explores the application of an explainable machine learning approach for diabetes prediction through a two-stage process. In stage one, five machine learning models were trained using a publicly available dataset of diabetes symptoms for early diabetes classification from the Kaggle platform. In stage two, the same models were trained and evaluated on a dataset collected by Community Health Workers (CHWs) across six districts in Rwanda. SHAP (Shapley Additive exPlanations) was employed in both stages to generate interpretable outputs, providing clinically relevant insights that enhance trust, transparency, and fairness in model interpretation, thereby supporting informed clinical decision-making. This study aims to develop a predictive tool for T2DM risk to support community health workers (CHWs) in identifying individuals at risk through a two-stage screening process. The remainder of the paper is organized as follows: Section 2 discusses related studies, Section 3 outlines the methodology employed in this study, Section 4 presents the experimental results, Section 5 discusses the findings, and Section 6 concludes the study and highlights future research related to this work.

## 2 Related studies

Early diabetes prediction using machine learning has gained attention in recent studies. However, the limited availability of comprehensive datasets constrains the generalizability of existing studies. A considerable number of prior works have primarily relied on the PIMA Indian Diabetes dataset, which includes 768 samples and 9 features, as well as the early-stage diabetes risk prediction dataset available on Kaggle, which includes 520 samples and 17 features.

In a study (12), three machine learning algorithms, Multilinear Regression (MLR), Random Forest (RF), and XGBoost, were employed to predict diabetes. The XGBoost model achieved a predictive accuracy of 99.23% using the complete dataset, which comprised 16 features and 1 target class. Notably, the same level of accuracy was achieved when only 9 features were utilized, selected based on feature importance rankings derived from the Random Forest model, using the early-stage diabetes risk prediction dataset.

Logistic Regression (LR), Naive Bayes (NB), Random Forest (RF), XGBoost, Support Vector Classifier (SVC), and Linear Discriminant Analysis (LDA) were used to predict diabetes using the PIMA Indian dataset (13). The SVC model scored the highest accuracy (82.5%). SHAP was used to provide the global model explanations, and Local Interpretable Model-agnostic Explanations (LIME) were used to explain the model's output at the individual patient level.

A good predictive performance was reported in (14), where researchers used Gaussian Naive Bayes, Random Forest, Support Vector Machine, Logistic Regression, and Decision Tree classifiers to predict diabetes using a dataset of 3,837 samples from Bangladeshi diabetes patients with 16 features and 1 class label, similar to the early-stage diabetes risk prediction dataset. The best model was Random Forest with an accuracy of 98%.

A hybrid deep learning model comprising a genetic algorithm, a stacked autoencoder, and a Softmax classifier was developed for diabetes prediction using the Early Stage Diabetes Risk Prediction dataset (15). The proposed model scored the highest performance with an accuracy of 98.72%. This model was compared with other models trained on the same dataset. These models include nearest neighbor, decision tree, support vector machine, and convolutional neural network, trained using tenfold cross-validation. The second-best model was CNN, with an accuracy of 96.15%.

Oladosu et al. (16) employed four machine learning models, including Random Forest (RF), Naïve Bayes (NB), KNN, and Decision Tree (J48), for the early detection of diabetes using a dataset of 520 samples and 16 predictors. A feature selection process was performed, during which three features (predictors) were eliminated due to their low information gain. All models were trained and evaluated using 520 samples and the remaining 13 features, and the Random Forest scored the highest accuracy of 98.30%. This study showed that polyuria and polydipsia are important predictors of diabetes risk.

## 3 Materials and methods

This study aimed to develop a T2DM risk prediction model using explainable machine learning in a two-stage approach. Five machine learning models, including Multi-Layer Perceptron (MLP), K-Nearest Neighbor (KNN), XGBoost, Support Vector Machine (SVM), and Naïve Bayes, are utilized for stages one and two. The selection of these models was based on their popularity and the learning techniques they employed. For each stage, the best model is explained using SHAP.

### 3.1 Datasets description

**Figure 1:**
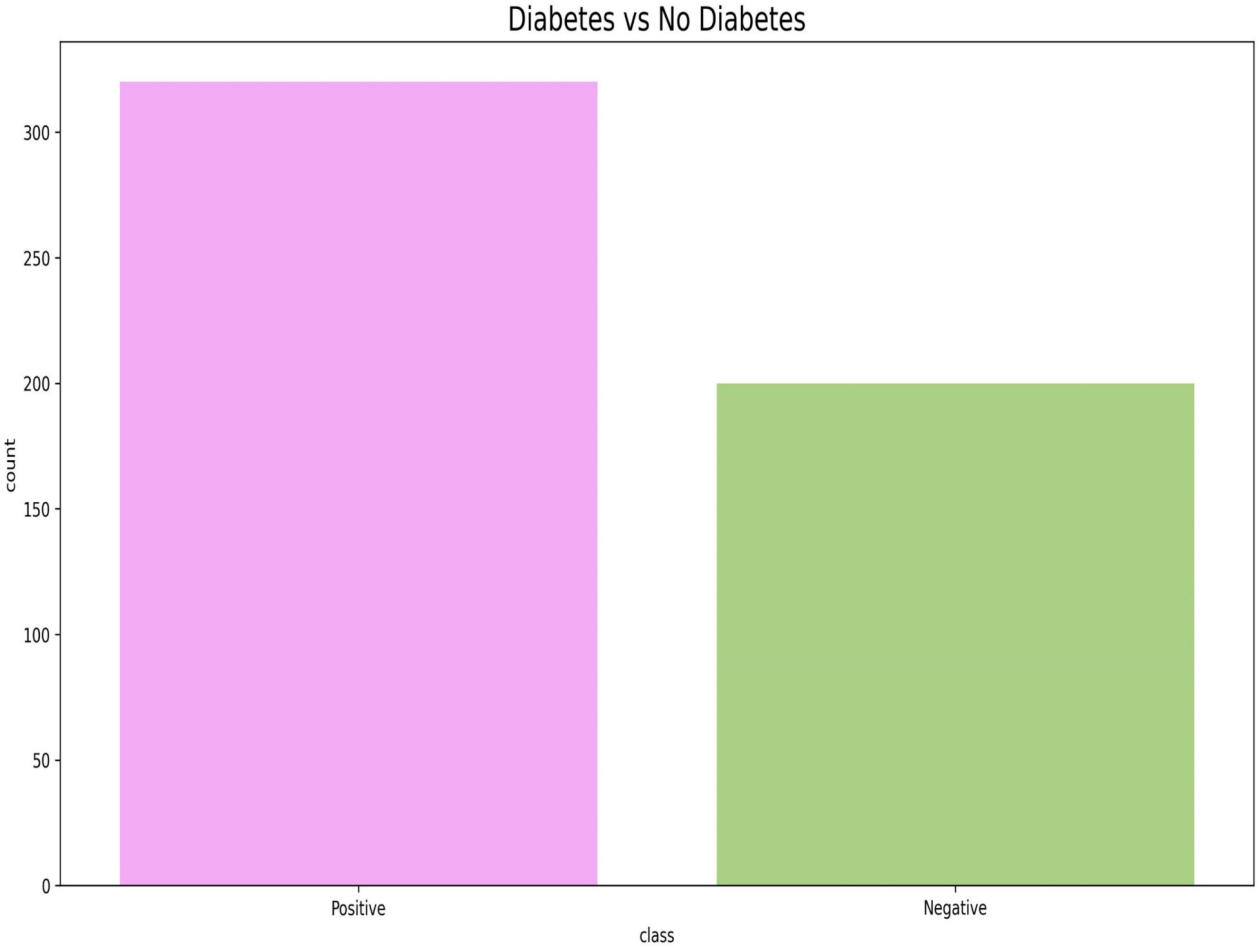
Class distribution of the public dataset for diabetes prediction.

In stage one of this study, an early diabetes classification dataset from Kaggle (17) was utilized. The dataset comprises 520 samples and 16 features, including Age, Gender, and 14 additional diabetes-related symptoms, as well as a target class. Table 1 describes the dataset used in stage one. As shown in Figure 1, the dataset includes 320 positive and 200 negative cases, indicating a relatively balanced class distribution.

**Table 1:** Description of the dataset used in stage one.

Feature Name	Data type	Value	Description
Age	Numeric	Numeric value	Demographics data: Min age = 16 years, Max age = 90 years
Gender	Binary	Male/Female	Demographics data: Male = 63%, Female = 37%
Obesity	Binary	Yes/No	Demographics data: Excess body fat
Polyuria	Binary	Yes/No	Clinical symptoms: Excessive production of urine, or excessive urination
Polydipsia	Binary	Yes/No	Clinical symptoms: Excessive thirst
Polyphagia	Binary	Yes/No	Clinical symptoms: Increased hunger
Sudden weight loss	Binary	Yes/No	Clinical symptoms: Unexplained rapid loss of body weight
weakness	Binary	Yes/No	Clinical symptoms: Decreased physical strength
Genital thrush	Binary	Yes/No	Clinical symptoms: Redness, itching, or painful genital organs due to yeast infection (common in diabetic patients)
Visual blurring	Binary	Yes/No	Clinical symptoms: Low vision due to high blood sugar levels
Delayed healing	Binary	Yes/No	Clinical symptoms: Wounds take longer to heal due to high blood sugar levels
Itching	Birary	Yes/No	Psychological signs: Dry skin becomes itchy due to elevated blood sugar
Irritability	Binary	Yes/No	Psychological signs: Change of mood due to blood sugar fluctuations
Partial paresis	Binary	Yes/No	Physical sign: Muscle weakness in some part of the body
Muscle stiffness	Binary	Yes/No	Physical sign: Reduced flexibility, tightness in the muscle
Alopecia	Binary	Yes/No	Physical sign: Hair loss
Class	Binary	Positive/ Negative	Classification: Diabetes (Positive), No Diabetes (Negative)

This dataset was also presented during a workshop held in Musanze District, Rwanda, from June 24–28, 2024, which brought together 18 participants, including nurses, doctors, data managers, and policymakers in healthcare technology, to discuss strategies for diabetes management using artificial intelligence. The participants have agreed that this dataset is suitable for an initial screening of T2DM using symptoms data and recommend reducing the feature set to the ten most relevant predictors of type 2 diabetes risk to minimize the time required for data entry by community health workers. Based on their experience in diabetes screening, they insisted that a reduced feature set be included, focusing on four symptoms (Polyphagia, Polyuria, Polydipsia, and Vision blurring), which are frequently encountered in the daily diagnosis process of diabetes.

Stage one of this study consists of diabetes risk screening based on diabetes symptoms and general health status. In stage two, individuals identified as higher risk undergo further assessment using demographic characteristics, anthropometric measurements, and vital sign parameters. Those who screen positive at both stages subsequently receive additional testing with a random or fasting blood glucose test, and if the test is positive, the patient is referred to the health center for further diagnosis. The final diagnosis is ultimately confirmed through a blood test that measures blood glucose levels, most commonly the HbA1C test.

**Figure 2:**
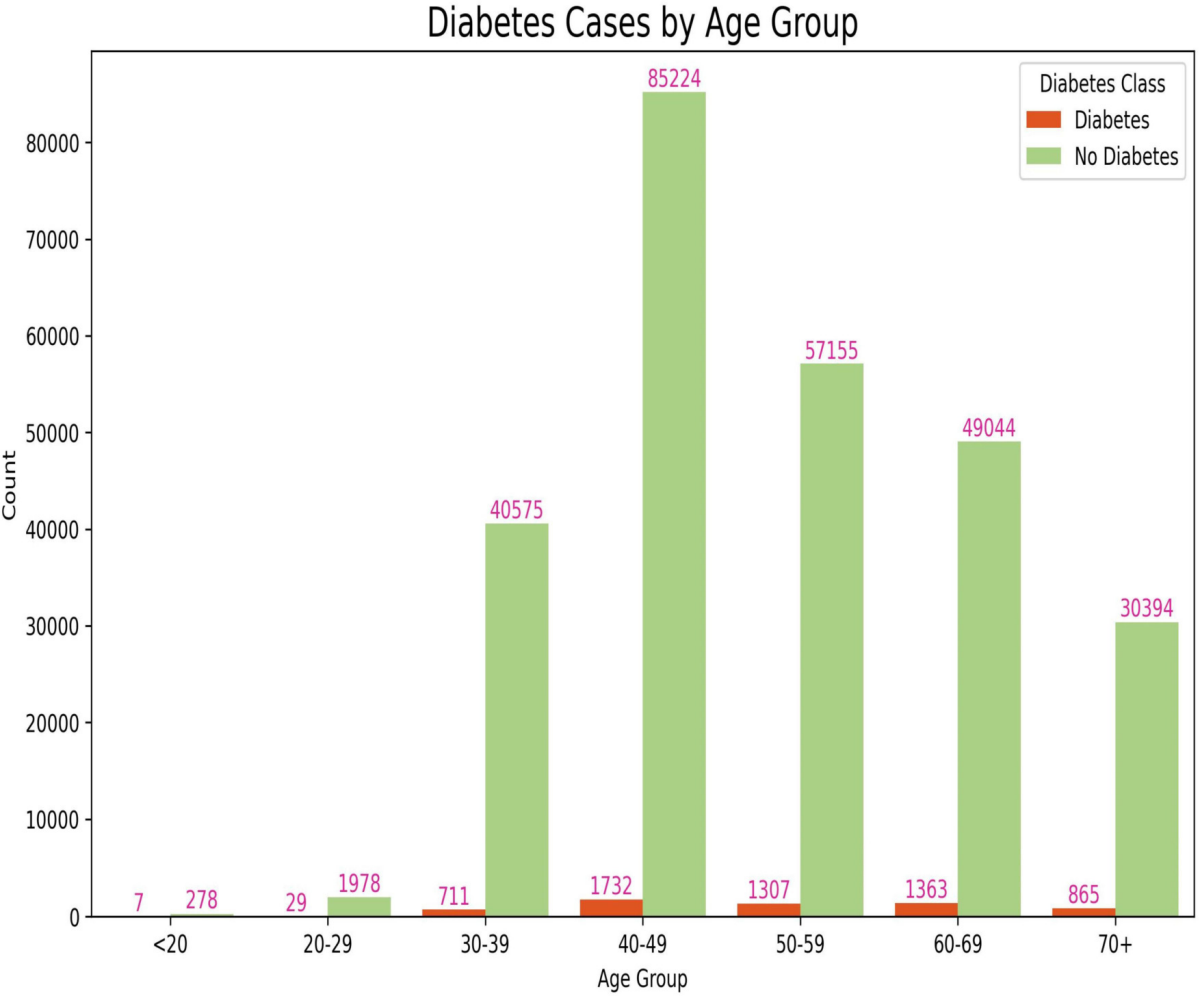
Class distribution by age group using a dataset from Rwanda.

Stage two of this study utilizes a dataset collected by community health workers across six districts in Rwanda using the mUzima mobile application. The dataset contains 270,943 records, each with 6 features: demographic information, anthropometric data, and vital signs data. The diagnosis label includes 6,022 diabetes risk cases and 264,921 non-diabetes cases, indicating a significant class imbalance. To address this, SMOTE (18) was used to balance the dataset. Figure 2 illustrates the distribution of diabetes cases across different age groups.

Community Health Workers (CHWs) initially assess diabetes risk using either a random or a fasting blood glucose test. The fasting glucose test is administered to individuals who have abstained from food and drink, except water, for at least eight hours prior to the test. A result is considered positive if the blood glucose level is 126 mg/dL or higher. The random blood glucose test, which can be conducted at any time, regardless of the last meal, is considered positive when the glucose level is 200 mg/dL or higher. Individuals identified as at high risk based on these preliminary tests are referred to health centers for further testing using the HbA1c (glycated hemoglobin) test, which reflects average blood glucose levels over the preceding three months. According to clinical guidelines, an HbA1c level of <5.7% indicates a non-diabetic status, 5.7%–6.4% indicates prediabetes, a stage at which intervention may prevent progression to diabetes, and a level of ≥6.5% confirms a diagnosis of diabetes.

### 3.2 Data preprocessing

**Figure 3:**
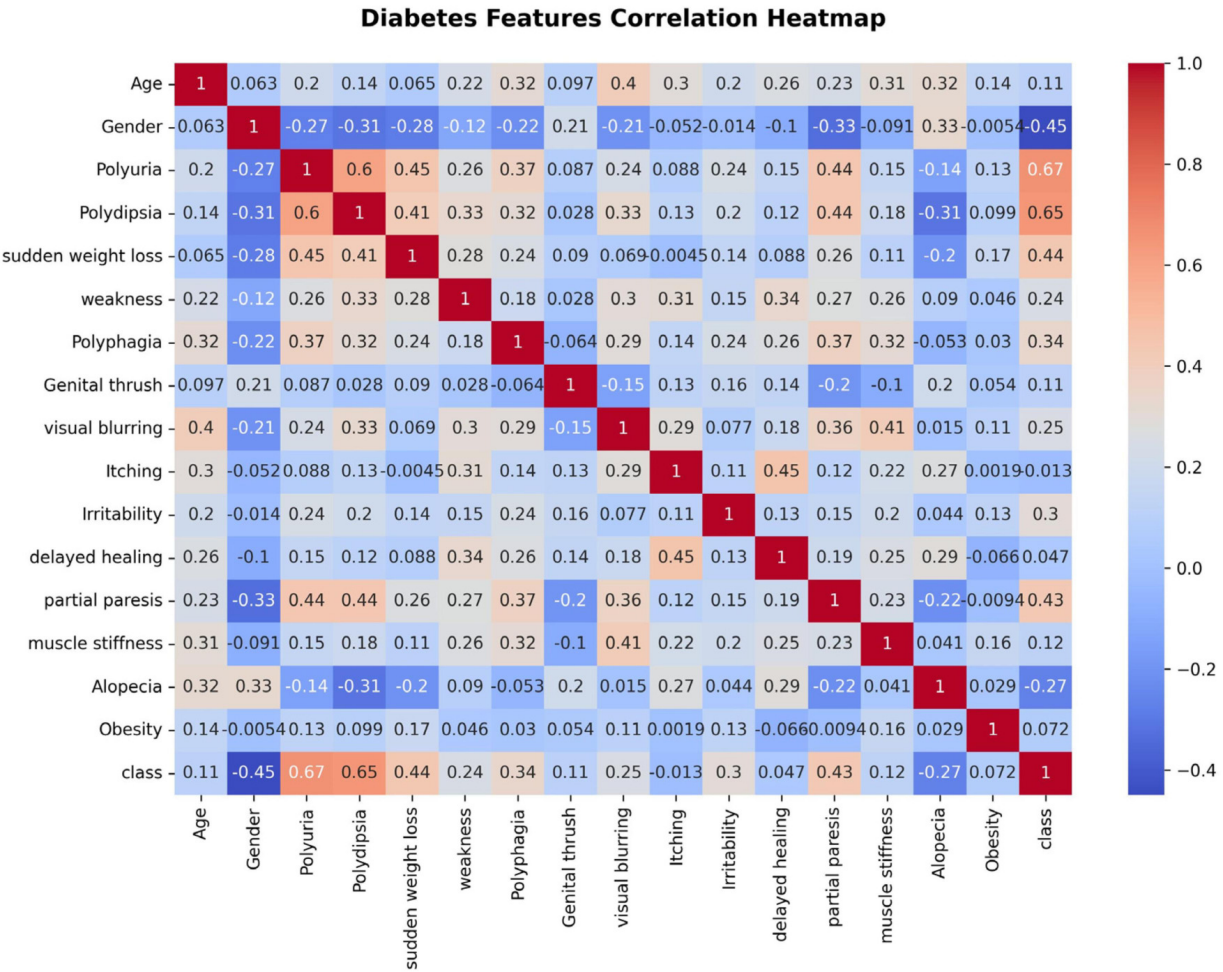
Features correlation to diabetes risk.

Data preprocessing is a critical step in preparing datasets for machine learning applications. The dataset used in stage one comprises one numerical feature (Age) and 15 binary categorical features. To enable model compatibility, categorical variables were converted into a numerical format using label encoding. The Age feature was standardized by applying Standard Scaler normalization. Figure 3 presents the correlation between the input features and the risk of diabetes.

The dataset used in stage one contains no missing values and exhibits a relatively balanced class distribution.

**Figure 4:**
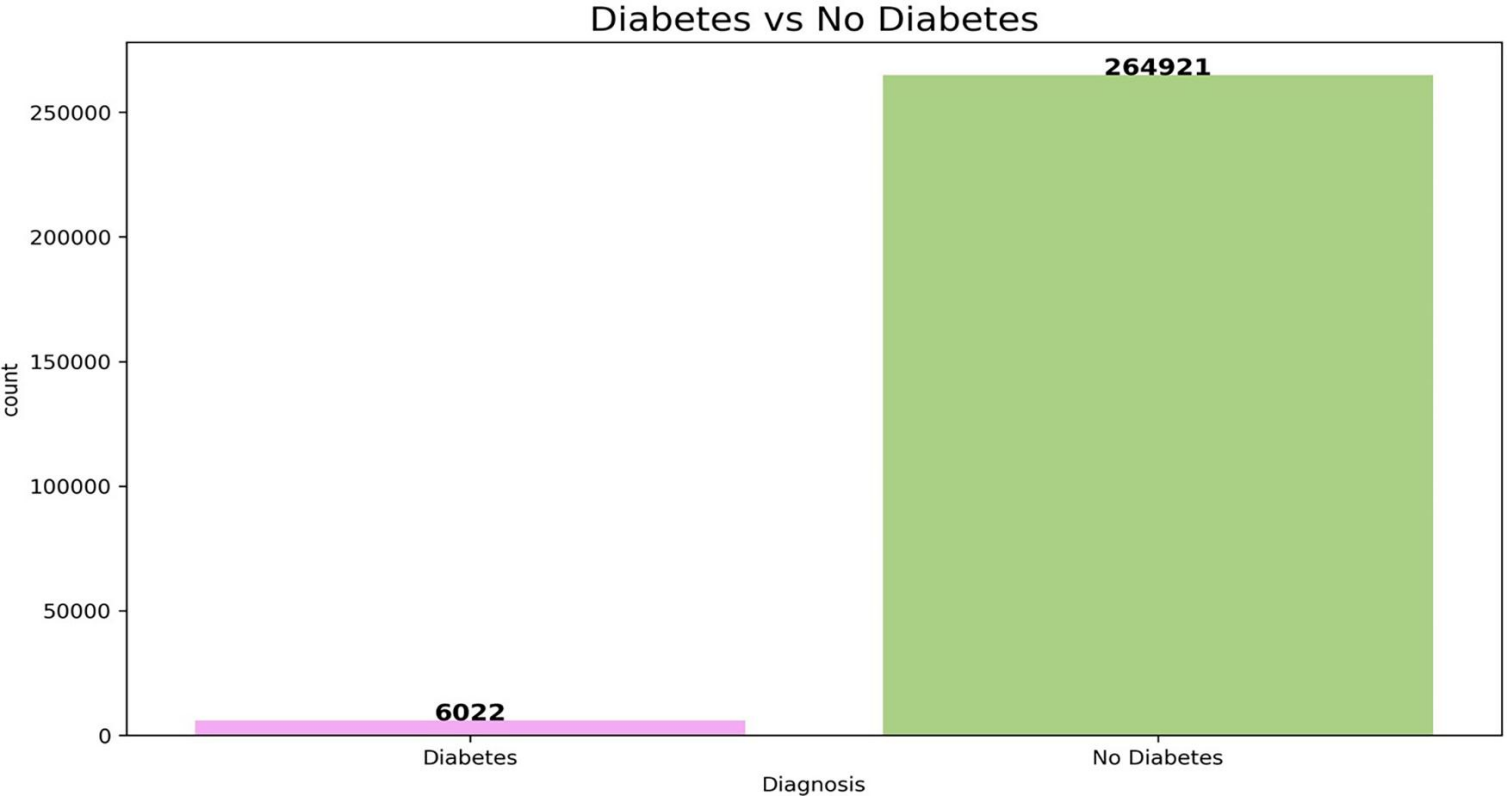
Class distribution of the dataset from Rwanda.

The dataset used in stage two has many features with higher rates of missing values. To maintain data integrity and minimize bias, features with more than 50% missing values were excluded from the analysis, as excessive dataset imputation could compromise the models' reliability. The final dataset comprises six features, including one binary categorical feature (Gender) and five numerical features. As glucose test methods and associated glucose measurements constitute the gold standard for determining diabetes status, they were deliberately excluded from the model development process, including training, validation, and testing phases. Incorporating these variables would introduce outcome leakage and circular reasoning, allowing the models to rely solely on predictors that directly define the target variable. Their exclusion ensured that the models learned meaningful patterns from independent risk factors. For the included numerical features, missing values were imputed using the mean. Figure 4 illustrates the class distribution of the dataset used in stage two of this study.

The dataset exhibits a significant class imbalance, with non-diabetic cases greatly outnumbering diabetic cases. To address class imbalance, the Synthetic Minority Over-sampling Technique was employed to enhance the representation of the minority class. To minimize the potential bias introduced by synthetic samples while maintaining class balance, SMOTE was applied exclusively to the training dataset (70%) and the validation dataset (10%) used for hyperparameter tuning. The test dataset (20%) was left unaltered to preserve the original class distribution and to ensure that model performance was evaluated under realistic clinical conditions, where positive cases are relatively rare. The optimal model configuration was determined using grid search with K-fold cross-validation. A relatively small number of folds (K = 3) was selected to reduce computational burden while maintaining reliable performance estimation across multiple splits. Each model was trained and hyperparameter-tuned independently, and their performance was systematically evaluated to enable consistent and transparent comparison across all candidate models.

### 3.3 Description of proposed approach for diabetes risk screening

**Figure 5:**
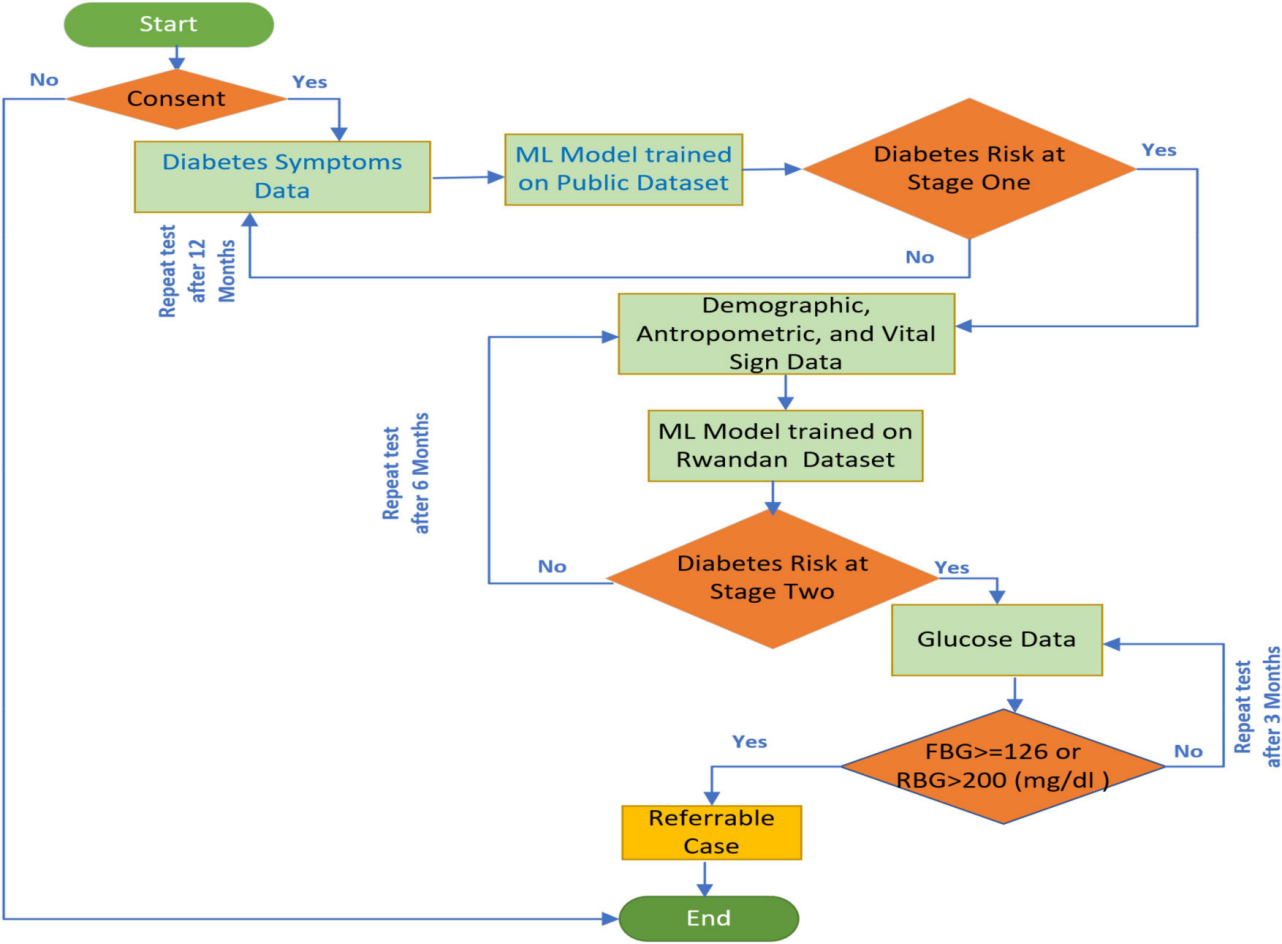
Workflow of the proposed AI-based diabetes screening.

This study aimed to develop and deploy a machine learning–based tool to automate diabetes risk detection across two sequential screening stages. In stage one, the model identifies individuals at potential risk of diabetes based on reported symptoms. In stage two, individuals classified as higher risk in stage one undergo further risk prediction for type 2 diabetes mellitus (T2DM) using demographic characteristics, anthropometric measurements, and vital sign data. The predictive models from both stages will be integrated into the mUzima application to support community health workers in accurately and efficiently screening for diabetes risk. Together, the two-stage approach is designed to reduce the burden of undiagnosed diabetes and promote the adoption of artificial intelligence–driven screening tools in routine care. Specifically, stage two facilitates early detection of T2DM risk among individuals flagged in stage one, enabling timely intervention and improved diabetes management at the health center level. The overall screening workflow of the proposed approach is illustrated in Figure 5.

### 3.4 Machine learning models

Five machine learning models, including MLP, KNN, XGBoost, SVM, and Naïve Bayes, were used for both datasets, and they were evaluated using the metrics defined by the following formulas:

$$\mathrm{ccuracy}=\frac{\mathrm{TP}+\mathrm{TN}}{\mathrm{TP}+\mathrm{TN}+\mathrm{FP}+\mathrm{FN}}$$

$$\mathrm{Precision}=\frac{\mathrm{TP}}{\mathrm{TP}+\mathrm{FP}}$$

$$\mathrm{Sensitivity}=\frac{\mathrm{TP}}{\mathrm{TP}+\mathrm{FN}}$$

$$\mathrm{Specificity}=\frac{\mathrm{TN}}{\mathrm{TN}+\mathrm{FP}}$$

$$\mathrm{Recall}=\frac{\mathrm{TP}}{\mathrm{TP}+\mathrm{FN}}$$

$$F1\text{-}\mathrm{score}=\frac{2*\mathrm{TP}}{2*\mathrm{TP}+\mathrm{FP}+\mathrm{FN}}$$

In this study, TP denotes true positives, TN denotes true negatives, FP denotes false positives, and FN denotes false negatives. In addition to conventional evaluation metrics, the Area Under the Precision–Recall Curve (AUPRC), also known as average precision, and the Area Under the Receiver Operating Characteristic Curve (ROC-AUC) were used to assess model performance. AUPRC is derived from precision and recall and specifically evaluates performance with respect to the positive class, incorporating TP, FP, and FN. To evaluate the models of stage one, Accuracy, Precision, Recall, F1-score, and AUC score are used, while Accuracy, Precision, Sensitivity, Specificity, and AUPRC score are used to compare model performance on predicting diabetes risk using demographics, anthropometric, and vital signs data. The metrics used to analyze the stage two models are less sensitive to class imbalance.

#### 3.4.1 Multi-Layer perceptron (MLP)

The Multi-Layer Perceptron (MLP), a type of Artificial Neural Network (ANN), is designed to learn complex patterns and non-linear relationships within data by iteratively updating weights and biases through a feedforward and backpropagation architecture. An MLP typically consists of an input layer, one or more hidden layers composed of interconnected computational units (neurons or nodes), and an output layer. In (19), an MLP was employed to predict the risk of diabetes.

#### 3.4.2 K-Nearest neighbor (KNN)

The K-Nearest Neighbor (KNN) algorithm is a straightforward yet effective non-parametric machine learning model that classifies data based on the proximity of neighboring data points. The parameter K represents the number of nearest neighbors considered in the decision-making process. Using a specified distance metric, KNN determines the class of a new data point by evaluating its closest neighbors. In (20), KNN was applied to predict the risk of diabetes.

#### 3.4.3 Extreme gradient boosting (XGBoost)

XGBoost (Extreme Gradient Boosting) is a supervised machine learning model that constructs decision trees sequentially, where each tree is trained to correct the errors made by its predecessor through gradient-based optimization. By incorporating regularization and advanced optimization techniques, XGBoost enhances predictive performance while minimizing training time. In the study (21), XGBoost achieved a predictive accuracy of 94% in predicting the risk of diabetes.

#### 3.4.4 Support vector machine (SVM)

The Support Vector Machine (SVM) is a machine learning model commonly used for classification and regression tasks. It operates by identifying a decision boundary that linearly separates the data. In cases where the data is not linearly separable, SVM constructs the decision boundary, or hyperplane, by mapping the data into a higher-dimensional space using the kernel trick. In (22), SVM was utilized to predict the risk of diabetes.

#### 3.4.5 Naïve Bayes

Naïve Bayes is a generative classification model that applies Bayes' theorem to make predictions based on conditional probability, under the assumption that features are conditionally independent. It estimates the probability of a class for new data points by considering the joint probability distribution of the input features. Despite its simplicity, Naïve Bayes has proven to be an effective approach. In (23), it was employed to predict diabetes risk, achieving an accuracy of 81%.

### 3.5 Explainable machine learning

Explainable Artificial Intelligence (XAI) is an emerging field that focuses on developing methods to interpret and understand the decision-making processes of machine learning models. In this study, we employed Shapley Additive exPlanations (SHAP) methods due to their proven effectiveness in generating human-interpretable insights. Specifically, Kernel SHAP was used in this study. Kernel SHAP (24) has a strong theoretical background rooted in cooperative game theory. It was applied to both datasets to identify the most influential features contributing to diabetes risk prediction. In stage one, SHAP was utilized as a feature selection technique to identify the top 10 features strongly associated with diabetes risk. In the Stage two dataset, SHAP was used to rank the contribution of all six features in terms of their impact on the model's predictions, as well as to interpret patient-level T2DM risk prediction.

## 4 Experimental results

In this study, diabetes prediction is formulated as a binary classification task. The selected machine learning models demonstrated the ability to accurately predict the risk of Type 2 Diabetes Mellitus (T2DM), with all models achieving an accuracy exceeding 70% in both stages.

### 4.1 Diabetes risk prediction stage One

Experimental results indicated that the Multi-Layer Perceptron (MLP) model outperformed the other models across three out of five performance metrics, achieving an accuracy of 95.19% in predicting diabetes risk using the public dataset. Table 2 presents a comparative summary of the performance metrics for all models.

**Table 2:** Models' performance on public dataset with all features.

Classifier	Accuracy	Precision	Recall	F1-Score	AUC
SVM	92.31	100.00	87.88	93.55	0.991
XGBoost	74.04	72.41	95.45	82.35	0.913
KNN	90.38	96.67	87.88	92.06	0.977
NaiveBayes	89.42	91.04	92.42	91.73	0.932
**MLP**	**95.19**	98.41	93.94	**96.12**	**0.998**

Bold values indicate where one model outperformed other models.

**Figure 6:**
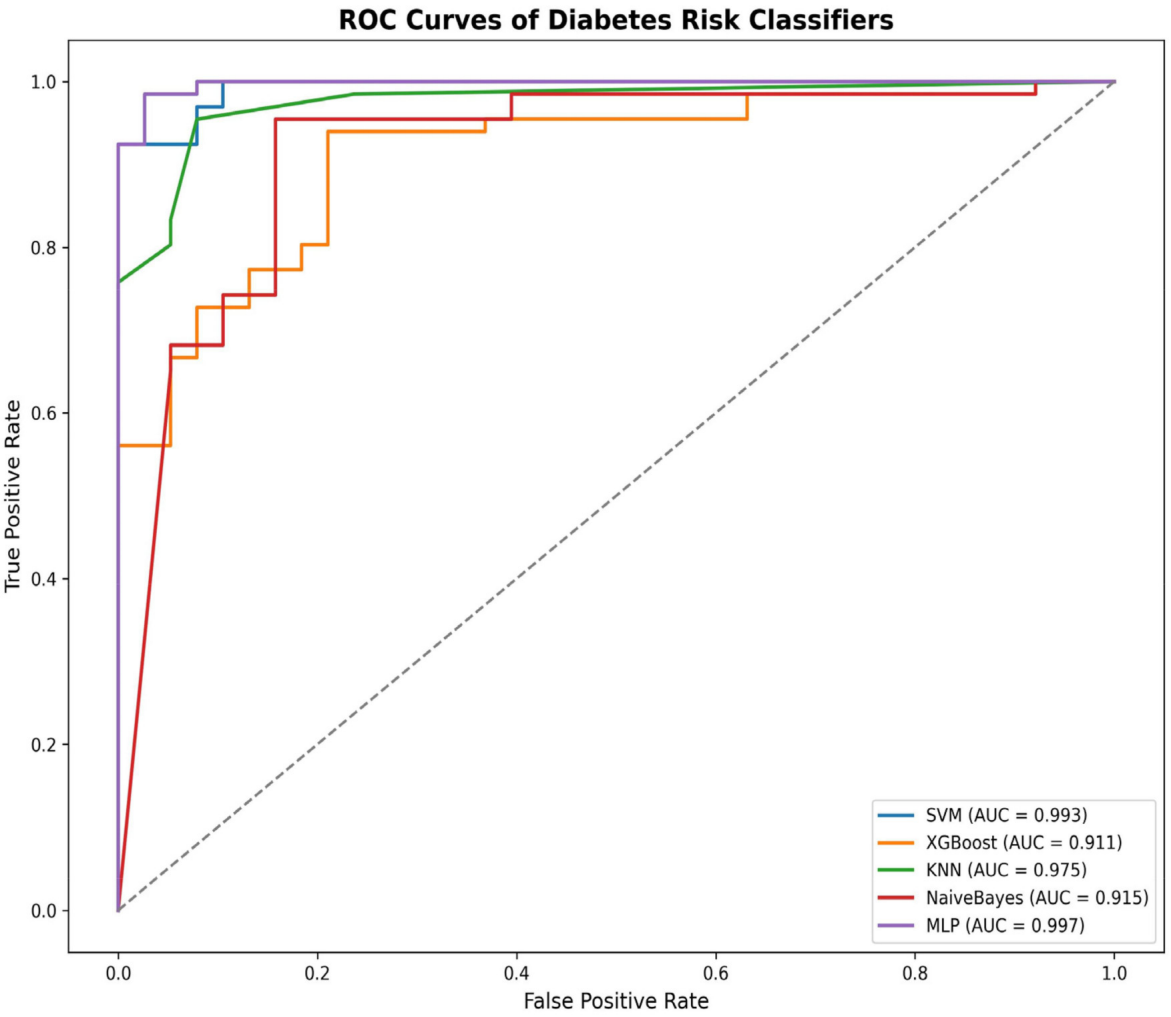
ROC-AUC curve of five classifiers on the public dataset.

Figure 6 presents the comparative performance of all evaluated models for diabetes risk prediction, as assessed by the ROC–AUC curve. Support Vector Machine (SVM) demonstrated the second-highest performance. In contrast, the XGBoost model achieved the lowest scores for accuracy, precision, and F1-score among the other models.

To respond to the request of diabetes care providers to reduce the feature set to the 10 most influential features for diabetes risk, we employed two methods, including SHAP and MLP model weights, to compute the feature importance for the diabetes prediction model.

#### 4.1.1 Experimental results of feature selection using SHAP methods

**Figure 7:**
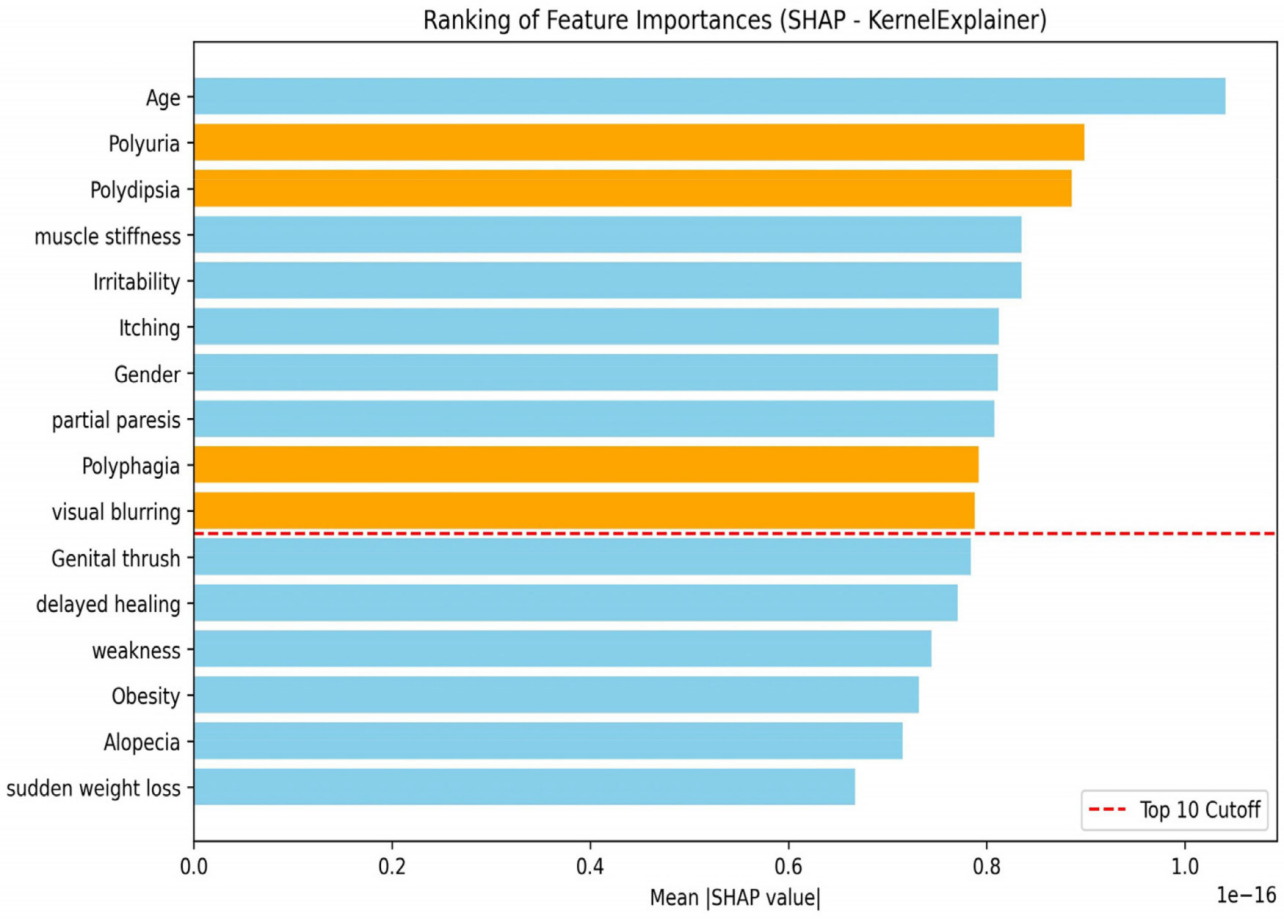
Feature importance visualization by SHAP methods.

Figure 7 highlights the feature ranking by SHAP methods. To evaluate the relevance of selected features, all the models were retrained on the dataset using the top ten features identified by SHAP, and the same performance metrics were used.

To emphasize the features that nurses might wish the model to consider, we used orange, and the red dashed line marks the ten most important features for diabetes diagnosis. Table 3 shows the models' performance on the reduced feature set of 10 features.

**Table 3:** Models' performance on the public dataset with 10 features selected by SHAP.

Classifier	Accuracy	Precision	Recall	F1-score	AUC
SVM	90.38	96.67	87.88	92.06	0.960
XGBoost	76.92	75.00	95.45	84.00	0.901
**KNN**	**94.23**	**95.45**	95.45	**95.45**	0.974
NaiveBayes	85.58	90.48	86.36	88.37	0.934
MLP	93.27	95.38	93.94	94.66	0.987

Bold values indicate where one model outperformed other models.

The KNN model demonstrated the highest performance on the reduced feature set with K = 5, achieving an accuracy of 94.23%. In general, all models showed improved performance after feature reduction using SHAP, except the MLP, which experienced a 1.92% decrease in accuracy and ranked second. These findings highlight the effectiveness of the SHAP-based feature selection method in identifying the most relevant predictors for assessing type 2 diabetes risk.

#### 4.1.2 Experimental results of feature selection using MLP weights

**Figure 8:**
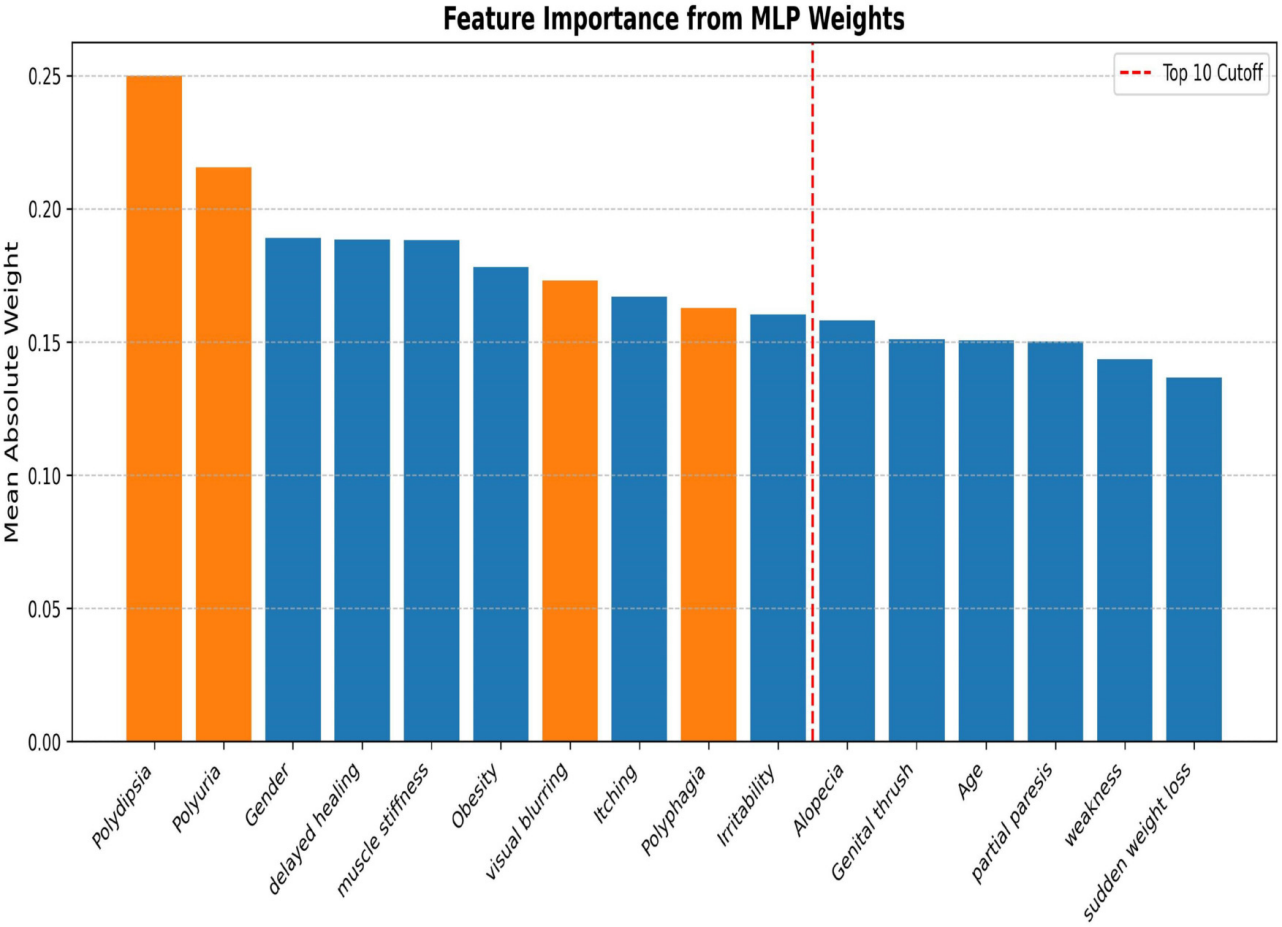
Feature importance ranking by MLP weights.

The second feature selection method computes feature importance by assigning weights to each feature during model building with the multi-layer perceptron (MLP) algorithm. Figure 8 illustrates the ranking of feature importance based on the MLP's assigned weights.

The MLP weight-based feature selection includes all four features, marking the increased risk of diabetes as suggested by the diabetes care providers during the workshop. Table 4 shows the performance of all models on the dataset with ten features selected by MLP.

**Table 4:** Models' performance on public dataset with 10 features selected by MLP.

Classifier	Accuracy	Precision	Recall	F1-score	AUC
SVM	93.27	98.36	90.91	94.49	0.984
XGBoost	76.92	73.86	98.48	84.42	0.903
KNN	95.19	98.41	93.94	96.12	0.988
NaiveBayes	82.69	88.71	83.33	85.94	0.941
MLP	**95.19**	96.92	**95.45**	**96.18**	**0.994**

Bold values indicate where one model outperformed other models.

The MPL model achieved superior performance, attaining an accuracy of 95.19% when trained and tested on a dataset with only 10 features highly correlated with diabetes risk. The second-best model is KNN with K = 5.

### 4.2 Diabetes risk prediction stage Two

In stage two of this study, the same five machine learning models used in stage one: Multi-Layer Perceptron (MLP), K-Nearest Neighbors (KNN), Support Vector Machine (SVM), XGBoost, and Naïve Bayes, were applied to a dataset collected in Rwanda. These models were trained and evaluated on a Rwandan dataset to assess their effectiveness in predicting type 2 diabetes risk in the local context. The performance outcomes of all models are summarized in Table 5. The experimental results demonstrate that each model achieved strong predictive performance, with all models attaining an accuracy exceeding 90%. This highlights the potential applicability of these machine learning approaches.

**Table 5:** Models' performance on the Rwanda dataset.

Classifier	Accuracy	Precision	Sensitivity	Specificity	AUPRC
SVM	95.13	69.34	98.32	94.75	0.94
**XGBoost**	**97.14**	**79.71**	98.50	96.97	**0**.**98**
KNN	96.60	79.09	93.08	97.03	0.64
NaiveBayes	91.69	56.88	94.70	91.33	0.959
MLP	95.66	71.65	98.80	95.28	0.96

Bold values indicate where one model outperformed other models.

**Figure 9:**
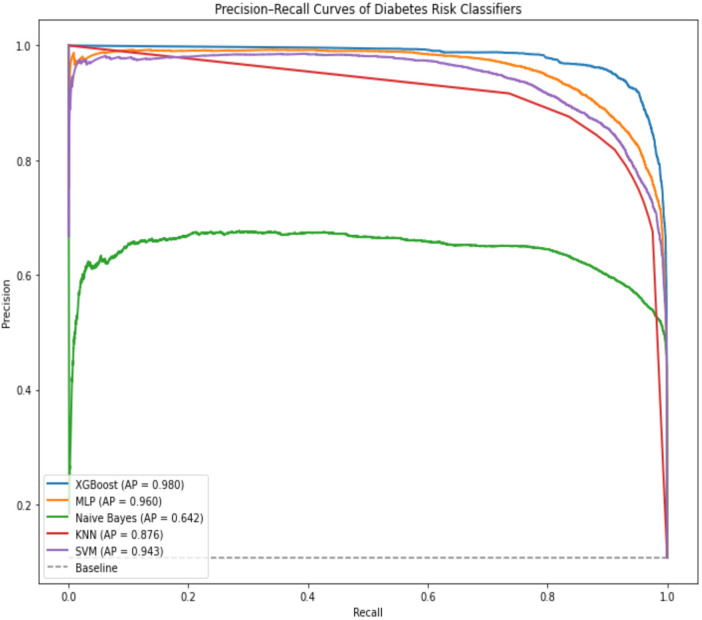
Precision-Recall curve of all models with their average precision (AP).

The XGBoost model outperformed all other models across three of five performance metrics, achieving an accuracy of 97.14%, followed by the KNN model, which attained 96.60%. Naïve Bayes scores the lowest performance with an accuracy of 91.69%. To deeply assess the performance of these models trained on the SMOTE training and validation datasets and evaluated on an unaltered test dataset (with class imbalance), Figure 9 compares them using the precision-recall curve.

The XGBoost model outperformed all other classifiers in identifying positive cases, achieving the highest average precision (AP = 0.98). In contrast, the Naïve Bayes model yielded the lowest average precision (AP = 0.64). Nevertheless, this performance remains notable given the pronounced class imbalance, with positive cases representing only 2.2% of the dataset.

### 4.3 Model explainability by SHAP

**Figure 10:**
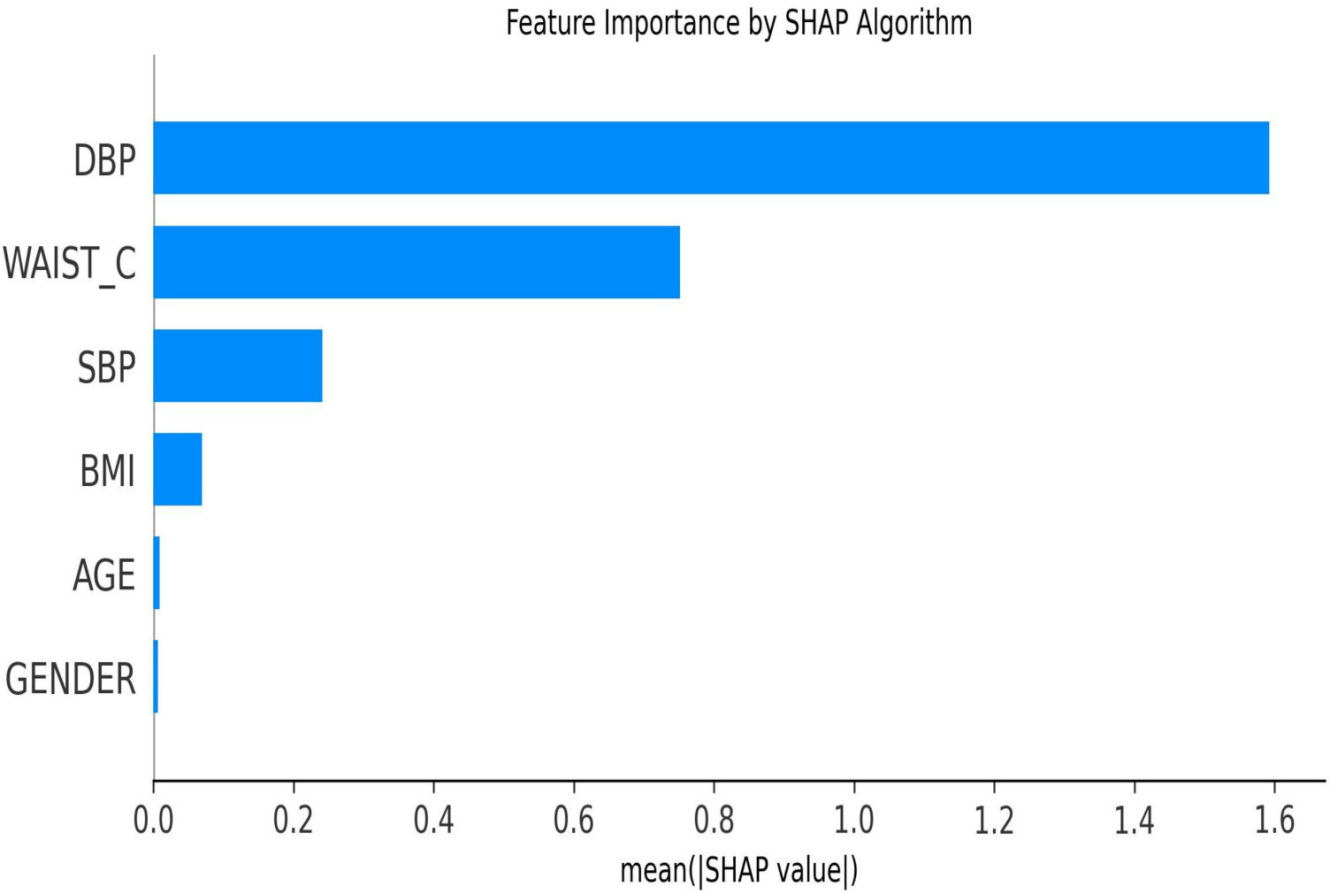
Diabetes risk prediction feature importance by SHAP.

In stage two of this study, SHAP was used to rank feature contributions to diabetes risk prediction. Figure 10 illustrates the order of feature importance in predicting diabetes risk using a dataset from Rwanda. The dataset used in stage two includes six features: Waist Circumference (WAIST_C), Age (AGE), Body Mass Index (BMI), calculated from weight and height, Systolic Blood Pressure (SBP), Diastolic Blood Pressure (DBP), and Gender (GENDER). SBP below 120 and DBP below 80 indicate normal blood pressure.

**Figure 11:**
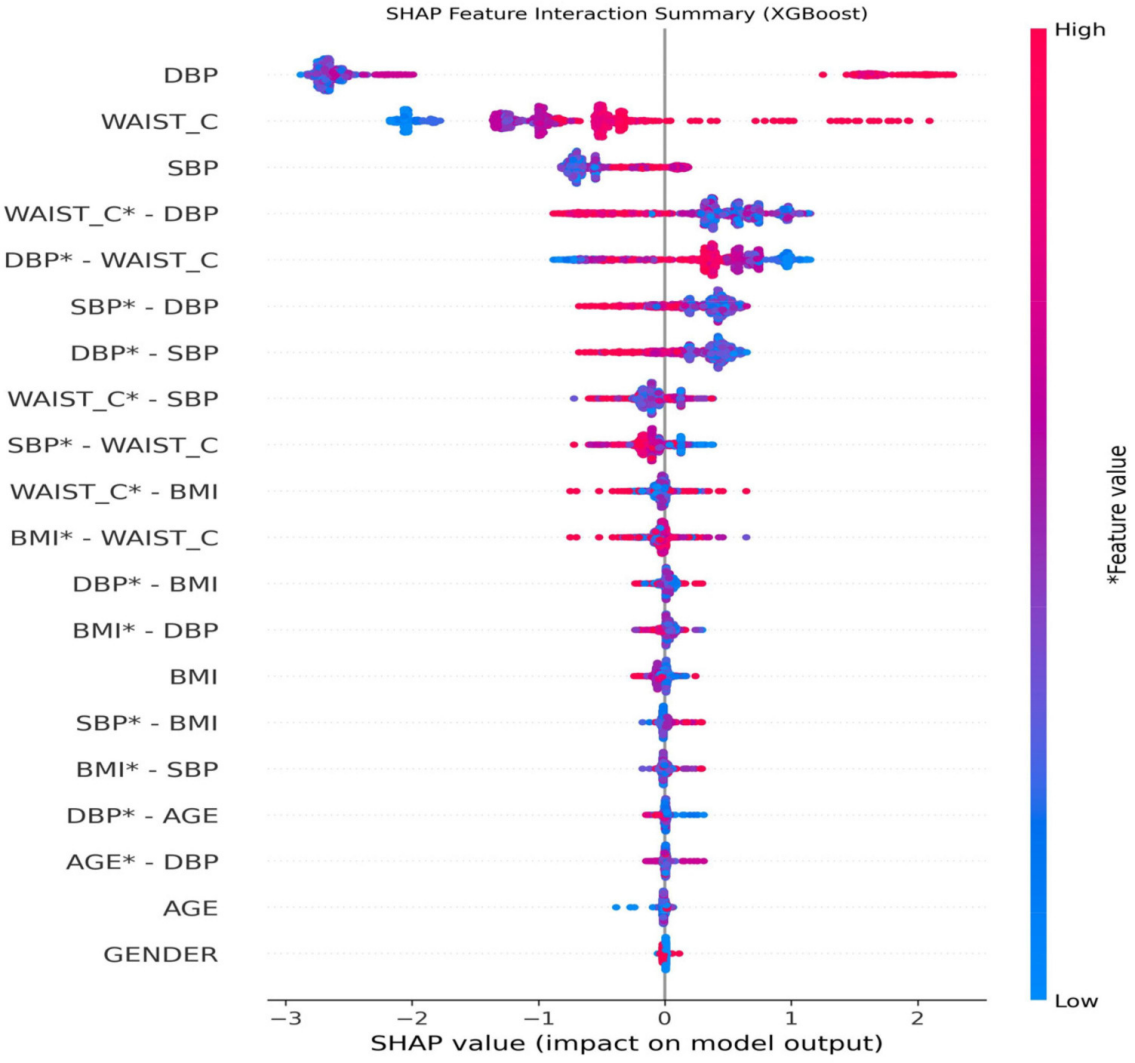
Diabetes risk prediction feature interactions by SHAP.

The feature importance plot highlights the potential influence of vital sign (DBP and SBP) and anthropometric (WAIST_C) features on the model decision when predicting diabetes risk. Among these predictors, DBP was ranked as the most influential feature, followed by WAIST_C. Figure 11 illustrates feature interactions in a global view, highlighting the contributions of individual features and of pairs of features to the diabetes risk prediction outcome.

**Figure 12:**
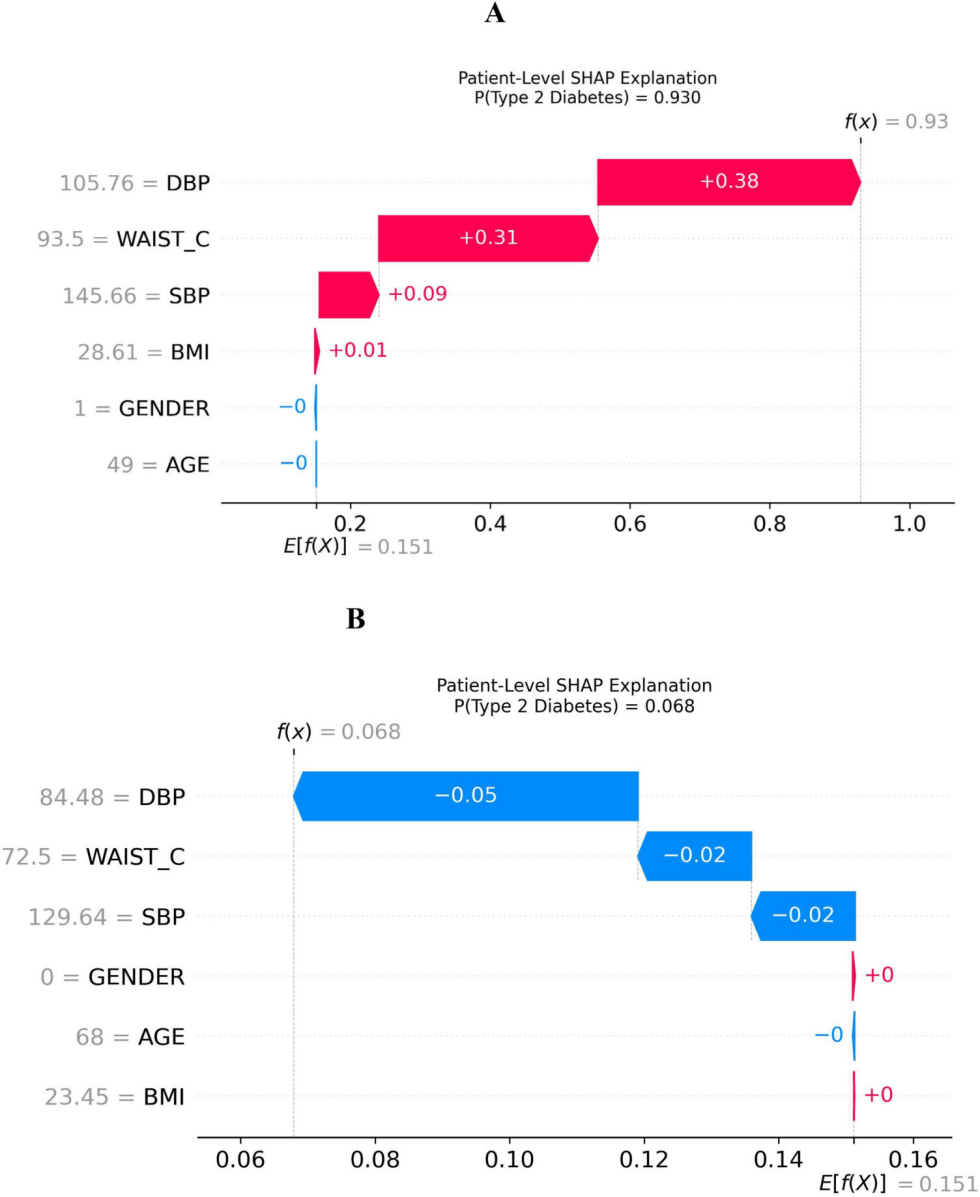
Patient-level model explanations by SHAP. **(A)** Positive case explanations by SHAP, **(B)** negative case explanations by SHAP.

The SHAP feature interaction summary plot indicates that age and gender have relatively limited influence on model predictions in stage two. In contrast, diastolic blood pressure (DBP) and waist circumference (WAIST_C) emerge as the most influential predictors of diabetes risk, followed by systolic blood pressure (SBP) and body mass index (BMI). Moreover, the interaction effects among these variables contribute substantially to the model's decision-making, underscoring their combined importance in risk stratification. The SHAP waterfall plot in Figure 12 explains the diabetes risk prediction for 2 individual patients: one has an increased risk of diabetes (Figure 12A), and the other has no risk of diabetes (Figure 12B) at stage 2 of this study.

Figure 12A illustrates a 49-year-old male patient whose clinical profile includes elevated BMI (28.61; normal: 18.5–24.9), diastolic blood pressure (105.76 mmHg; normal: 60–80), systolic blood pressure (145.66 mmHg; normal: 80–120), and waist circumference (93.5 cm; normal for males: <94 cm). The presence of these abnormal or borderline-high values explains the model's high predicted probability of type 2 diabetes risk (0.93), as indicated by the XGBoost output f(x) on the figure. In contrast, Figure 12B presents a 68-year-old female patient with clinical measurements closer to normal ranges, including BMI (23.45), DBP (84.48 mmHg), SBP (129.64 mmHg), and waist circumference (72.5 cm; normal for females: <80 cm). Based on these values, the model predicted that this patient is not at risk of type 2 diabetes as indicated by a probability of 0.068 on the figure.

## 5 Discussion

### 5.1 Key findings

The MLP model achieved superior performance, with an accuracy of 95.19% for predicting diabetes risk at stage one using the public dataset, while XGBoost excelled with an accuracy of 97.14% for predicting diabetes risk at stage two using a dataset from Rwanda. SHAP methods were used to identify the top 10 features contributing to diabetes risk prediction at stage one. Table 6 presents a description of selected features using the two-feature selection methods. In this study, the MLP architecture consisted of an input layer with 16 neurons (one per feature), a single hidden layer with 100 neurons using the ReLU activation function, and an output layer with one neuron employing a sigmoid activation function for binary diabetes classification (1 = Diabetes, 0 = No Diabetes). The model was trained with a learning rate of 0.001 and a batch size of 32.

**Table 6:** T2DM feature ranking by the two feature selection methods.

Ranking	Features selected by SHAP	Features selected by MLP Weights
1	Age	Polydipsia
2	Polyuria	Polyuria
3	Polydipsia	Gender
4	Muscle stiffness	Delayed healing
5	Irritability	Muscle stiffness
6	Itching	Obesity
7	Gender	Visual blurring
8	Partial paresis	Itching
9	Polyphagia	Polyphagia
10	Visual blurring	Irritability

Both feature selection methods have identified the four recommended features linked to diabetes risk among the top ten features, and they agree on 8 features out of 10, with a different order of ranking; they disagree only on 4 features: SHAP includes “Age’ and “Partial paresis', and MLP includes “Delayed healing” and “Obesity”. The top two features selected by the MLP weights are highly correlated with diabetes risk, as found in (16). When a person has an excessive amount of urine produced and excessive thirst resulting in dry mouth and the need to drink every time, it is an indication that the person is at risk of developing diabetes or already has undiagnosed diabetes. Feature selection using MLP model weights identified the most relevant features, as the best model on a full feature set achieved a similar performance on a reduced feature set.

The XGBoost model outperformed other models in predicting diabetes risk using a dataset from Rwanda, achieving an accuracy of 97.14%, indicating the effectiveness of the SMOTE technique for balancing the dataset. Table 7 shows the model configuration after the parameter tuning using a validation dataset grid search with 3-fold cross-validation.

**Table 7:** Model parameter tuning.

No.	Classfier	Parameters tuned and values
1	XGBoost	learning_rate = 0.05, max_depth = 7, n_estimators = 400
2	MLP	Alpha = 0.05, hidden_layer_sizes = 100
3	SVM	C = 10
4	K-NN	K = 10
5	Naïve Bayes	No parameter tuned

### 5.2 Comparison of the developed model with state-of-the-art models

The authors in (25) have employed the XGBoost algorithm to predict diabetes risk using the Behavioral Risk Factor Surveillance System (BRFSS) dataset, which comprised 253,680 samples and 21 features. The model achieved an overall accuracy of 86.6%. However, performance on other evaluation metrics was comparatively lower, with a precision of 54.1%, a recall of 17.0%, and an F1-score of 25.9%, indicating limited effectiveness in identifying positive cases. Recursive feature elimination was applied to reduce the feature set to 15 predictors, and the model demonstrated similar performance on the dataset with 15 predictors. Additionally, SHAP provided both global and local interpretability, offering insights into the model's overall decision-making process and individual-level predictions.

XGBoost and CatBoost models were used in (26) to predict diabetes risk using a dataset collected in Iran comprising 3,203 samples (402 positive cases) and 80 features. Model performance was evaluated on both the original dataset and a modified version augmented with synthetic samples generated by SMOTE to increase representation of the minority (positive) class. The data were split into 80% for training and 20% for testing. Among the evaluated models, XGBoost demonstrated superior performance and was further optimized using randomized search with 10-fold cross-validation. The optimized model achieved 96.07% accuracy, 96.1% F1-score, and 99.29% AUC, indicating excellent predictive performance. Notably, SMOTE was applied to the entire dataset, which may have contributed to the reported high performance across all evaluation metrics. Furthermore, SHAP was employed to identify key predictors of type 2 diabetes risk and to provide patient-level interpretability of the model's predictions.

An Explainable AI Framework was developed in (27) for diabetes risk prediction using a relatively small dataset comprising 520 samples and 17 features, similar to the dataset employed in stage one of our study. To address class imbalance, SMOTE was applied to the training (80%) dataset, and the test (20%) subset was left unaltered. The framework incorporated multiple classifiers, including AdaBoost, Logistic Regression, and CatBoost, and was designed to operate in an online environment. Among the evaluated models, CatBoost achieved the best performance, with an AUC score of 0.99. Model interpretability was provided using SHAP, which was used to assess global feature importance and to explain individual predictions. Gender-specific analyses indicated that the top two predictors were polyuria and polydipsia for males, and polyuria and alopecia for females. Furthermore, the framework integrated a chatbot powered by large language models (LLMs) to respond to patient inquiries related to diabetes, thereby enhancing user interaction and accessibility.

The proposed model demonstrates performance comparable to existing approaches while introducing methodological novelty through its two-stage risk-prediction framework and clinically aligned SHAP-based explanations. Unlike single-stage models, the proposed approach mirrors the real-world workflow of diabetes screening, thereby enhancing its practical applicability. The SHAP explanations are consistent with established clinical reasoning, strengthening trust and interpretability in decision-making. Integration of the proposed model into the mUzima application used by community health workers (CHWs) in Rwanda (as illustrated in Figure 5) has the potential to substantially improve community-level diabetes screening and reduce the burden of undiagnosed cases. Given that each CHW is responsible for approximately 180–300 households, an automated and explainable screening tool can significantly enhance efficiency and early detection.

Under the proposed workflow, individuals identified as not at risk in stage one will undergo rescreening after 12 months. Those classified as high risk in stage one will proceed to stage two for further risk assessment using demographic, anthropometric, and vital sign data. If stage two also indicates high risk, confirmatory glucose testing will be performed using either random blood glucose (RBG; positive if >200 mg/dL) or fasting blood glucose (FBG; positive if ≥126 mg/dL). Individuals with positive results will be referred to a health center for further diagnostic evaluation, including glycated hemoglobin (HbA1c) testing. If RBG or FBG results are negative, repeat testing will be conducted after three months to account for physiological changes in glucose metabolism. If the stage two model predicts no diabetes risk and SHAP explanations support a low-risk assessment, rescreening will be scheduled after six months. The proposed explainable AI (XAI)-based screening system will be integrated with the OpenMRS platform to securely store patient records and prediction outputs, ensuring continuity of care. Ethical considerations are addressed by obtaining informed consent from individuals prior to participation in diabetes screening, thereby aligning the system with responsible AI and clinical practice standards.

### 5.3 Limitations of this study

This study has several limitations. The models developed in stage one were trained on a relatively small dataset, which may increase the risk of overfitting and limit generalizability. Although stage two models were trained on a larger dataset, the pronounced class imbalance required the application of SMOTE, which may have introduced bias into the training data. Additionally, the relatively limited number of features available in stage two may have constrained the models' ability to fully capture the underlying predictors of diabetes risk. Furthermore, the successful real-world implementation of the proposed framework will depend on adequate training for community health workers to effectively use the two-stage, explainable machine-learning–based diabetes screening approach.

## 6 Conclusion and future work

This study employed a two-stage approach to train and evaluate five machine learning models for predicting diabetes risk. The MLP demonstrated superior performance in predicting type 2 diabetes risk in stage one with an accuracy of 95.19%. The model for this stage is designed to predict type 2 diabetes risk based on symptoms. Using SHAP methods, the top ten most influential features were identified. Retraining all models with only these features yielded an accuracy of 95.19%, which is the same as that achieved by the MLP model when trained on all features, indicating the effectiveness of the feature selection methods employed.

In stage two, the XGBoost model achieved an accuracy of 97.14% in identifying referable cases of diabetes using data collected using the mUzima application. SHAP analysis provided global and local model explanations, ranking diastolic blood pressure as the most critical predictor, followed by waist circumference. Based on these findings, we recommend integrating the models of both stages into the mUzima app to support community health workers in efficiently identifying high-risk individuals. Furthermore, we encourage the use of comprehensive data recording to enhance model performance through richer feature sets, including socio-economic features.

Future work will focus on deploying stage one and stage two into the mUzima mobile application and integrating them with OpenMRS to support the identification of referable diabetes cases. Additionally, incorporating diabetes diagnoses using explainable machine learning into mUzima by health centers will enable continuous model updates and improvement.

## Data Availability

The datasets and the code for this study are available in the associated GitHub repository: https://github.com/sima1615/Early-Type-2-Diabetes-Risk-Prediction. Further inquiries can be directed to the corresponding author/s.
